# GRP78 at the Centre of the Stage in Cancer and Neuroprotection

**DOI:** 10.3389/fnins.2017.00177

**Published:** 2017-04-05

**Authors:** Caty Casas

**Affiliations:** Department of Cell Biology, Physiology and Immunology, Institut de Neurociències, Universitat Autònoma de BarcelonaBarcelona, Spain

**Keywords:** GRP78, BiP, neuroprotection, endogenous mechanisms, neurodegeneration, ER stress, autophagy, ERAD

## Abstract

The 78-kDa glucose-regulated protein GRP78, also known as BiP and HSP5a, is a multifunctional protein with activities far beyond its well-known role in the unfolded protein response (UPR) which is activated after endoplasmic reticulum (ER) stress in the cells. Most of these newly discovered activities depend on its position within the cell. GRP78 is located mainly in the ER, but it has also been observed in the cytoplasm, the mitochondria, the nucleus, the plasma membrane, and secreted, although it is dedicated mostly to engage endogenous cytoprotective processes. Hence, GRP78 may control either UPR and macroautophagy or may activated phosphatidylinositol 3-kinase (PI3K)/AKT pro-survival pathways. GRP78 influences how tumor cells survive, proliferate, and develop chemoresistance. In neurodegeneration, endogenous mechanisms of neuroprotection are frequently insufficient or dysregulated. Lessons from tumor biology may give us clues about how boosting endogenous neuroprotective mechanisms in age-related neurodegeneration. Herein, the functions of GRP78 are revealed at the center of the stage of apparently opposite sites of the same coin regarding cytoprotection: neurodegeneration and cancer. The goal is to give a comprehensive and critical review that may serve to guide future experiments to identify interventions that will enhance neuroprotection.

Several systems, including the nervous system, have a remarkable ability for repair under stressful conditions. Conserved intrinsic mechanisms counteract damaging effects of endogenous and/or exogenous toxic agents. Under circumstances of damage, intrinsic pro-survival pathways, that collectively are termed endogenous neuroprotective mechanisms, are activated. Endogenous protective mechanisms have been mainly investigated in diverse pathological states such as vascular diseases, trauma, and cancer. The question of why neurodegenerative diseases occur even when beneficial mechanisms have been triggered deserves in-depth analysis. GRP78 appears to orchestrate several of these endogenous mechanisms. We herein describe the characteristics and known functions of GRP78, explore its roles in tumor cell survival, proliferation, and chemoresistance and reflect on how this knowledge should guide investigations into its functions in neuroprotection.

## GRP78, a very important protein with multiple functions in multiple locations

### Transcriptional and post-translational regulation of GRP78 levels

**Figure 1:**
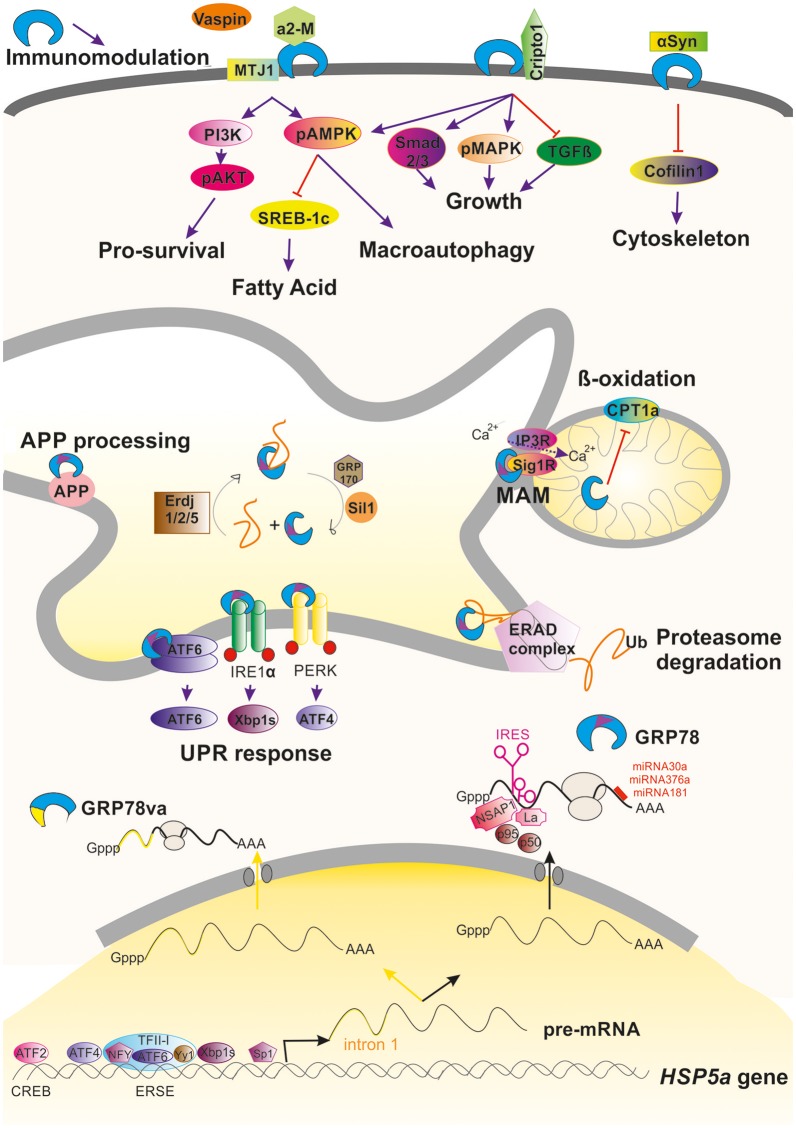
**Graphical summary of the regulation and activities promoted by GRP78 within a cell**. Induction and regulation of the transcription of the *HSP5a* gene is mediated by several transcription factors that bind to ERSE or CREB motifs in the promoter of the gene. Alternative processing of its pre-mRNA can occur under stressful conditions leading to retention of intron 1 (yellow line) that advance an stop codon, giving to GRP78va truncated protein that is retained in the cytosol because it lacks the ER-signaling motif (purple triangle). Commonly processing GRP78 is submitted under post-transcriptional regulation either due to the action of factors on its IRES motif or by the action of different miRNAs. GRP78 is found mainly in the luminal ER where it can promote the activation of the UPR, ERAD, or MAM regulation. In some circumstances, GRP78 can be translocated to the cell surface where it can interact to multiple partners and hence modulate different pathways. It is also be secreted where it can immunomodulate.

GRP78 has multiple functions in maintaining cell viability. Its expression is highly regulated at different points. At the transcription level, GRP78 is encoded by the gene *Hsp5a*. It is the most abundant protein within the heat shock protein-70 (Hsp70) family, but, unlike the other members of this family, it is not induced by heat shock because the promoter of *GRP78* lacks the heat shock element. Levels of GRP78 are maintained at relatively low levels within the cell and are increased considerably under stresses that affect the endoplasmic reticulum (ER) and calcium homeostasis. Indeed, GRP78 was initially discovered in 1977 as a 78-kDa protein strongly induced in chicken embryo fibroblasts cultured in glucose-free medium (Shiu et al., 1977). Later, it was observed that *GRP78* expression can be induced by other stimuli such as calcium ionophore A23187 (Resendez et al., 1985), calcium depletors or chelators such as thapsigargin and BAPTA-AM (Suzuki et al., 1991), and inhibitors of the protein secretory pathway such as tunicamycin (Lee, 1987). The upregulation of GRP78 expression under such a variety of stressful stimuli is mainly due to the presence of conserved elements in the promoter of the *Hsp5a* gene (Li and Lee, 2006) such as a CCAAT box (Resendez et al., 1988), a cAMP responsive element CRE-like (CREB; Alexandre et al., 1991), and the ER stress response element (ERSE; Resendez et al., 1988). Transcription factors that bind to these regulatory elements, including CBF/NF-Y (Roy and Lee, 1995), CREB, activating transcription factor 2 (ATF-2; Chen et al., 1997), YY1, YB1, Sp1 (Li et al., 1997), ATF4 (Luo et al., 2003), TFII (Parker et al., 2001), ATF6 (Yoshida et al., 2001b), and XBP1 (Yoshida et al., 2001a), participate in the regulation of *Hsp5a* gene (Figure 1).

The post-transcriptional regulation of GRP78 is mediated by the activation of internal ribosome entry sequence (IRES) in the 5′ untranslated region of *GRP78* mRNA (Macejak and Sarnow, 1991). IRESs are often present in mRNAs that encode proteins crucial for cell survival and stress recovery. Thus, in circumstances where repression of global protein synthesis is promoted, *GRP78* mRNA is selectively translated (Yang and Sarnow, 1997). In some situations, the presence of the IRES serves to amplify translation of *GRP78* mRNA. For instance, after infection of foreskin fibroblasts with human cytomegalovirus, activation of the *GRP78* IRES by the viral machinery results in a 3–4-fold increase of at the mRNA level but about a 50-fold increase at the protein level (Buchkovich et al., 2010). Other viral infections, including herpes simplex virus type 1 and poliovirus, have also been reported to activate the *GRP78* IRES (Kim et al., 2001; Saffran et al., 2010). Several cellular proteins are implicated in the translational activation of the GRP78 IRES including NS1-associated protein NSAP1, SSB/La autoantigen, p50, and p95 (Yang and Sarnow, 1997; Kim et al., 2001; Cho et al., 2007; Figure 1).

Another post-transcriptional regulatory mechanism acts on protein stability. It has been shown that activation of PI3K/AKT pathway in ER-stressed HEK-293 cells leads to an increase in GRP78 protein stability through unknown mechanisms (Dai et al., 2010). Regulation is also mediated through the action of specific microRNAs (miRNAs) such as miR-181 (Ouyang et al., 2012), miR-181a (Ji et al., 2017), miR-181b (Peng et al., 2013), miR-376a (Iwamune et al., 2014), and miR-30a (Wang P. et al., 2015) that bind to the *GRP78* mRNA 3′-untranslated region (Figure 1).

### GRP78 localization reflects multiple functions

GRP78 acts as a molecular chaperone (Haas and Wabl, 1983) and binds to nascent polypeptides. Like cytosolic HSP70, it contains an N-terminal ATPase domain and a C-terminal peptide binding domain (Määttänen et al., 2010). GRP78 is also a calcium binding protein. It is inhibited by a high concentration of calcium ions, and its ATPase activity is activated by calcium depletion. Due to the presence of an ER signaling peptide, GRP78 is mainly found in the ER lumen, although under some circumstances it is redistributed to the cytosol, nucleus, mitochondria, or the plasma membrane or is secreted (Suzuki et al., 1991). Thus, different locations prime GRP78 to trigger different molecular signaling events.

#### GRP78 multifunction associated with the endoplasmic reticulum

At the ER, GRP78 has diverse functions and relies on a number of interaction partners and co-chaperones, nucleotide exchange factors, and signal transducers for its various activities. The diversity of functions include translocating nascent polypeptides, facilitating *de novo* protein folding and assembly, targeting misfolded proteins to endoplasmic-reticulum-associated protein degradation (ERAD) machinery, and maintaining calcium homeostasis (since it is as a luminal calcium ER binding protein; Gardner et al., 2013). GRP78 is usually the first chaperone to bind a nascent polypeptide chain and prefers to bind surfaces with alternating aromatic and hydrophobic amino acids. GRP78 shifts to its tighter affinity substrate binding conformation after ATP hydrolysis to ADP (Blond-Elguindi et al., 1993). Several partners participate in this process. The hydrolysis of ATP by GRP78 is stimulated by ER resident J-domain co-chaperones (ERdj), ERdj1 and 2, homologs of yeast Sec63 (Otero et al., 2010), and also to co-chaperones such as P58(IPK) (Tao and Sha, 2011). In addition, the ADP-bound closed state of GRP78 is re-opened by exchange of ADP for ATP, and this process is enhanced by the nucleotide exchange factors GRP170 and Sil1, also known as BiP-associated protein (BAP; for a review see Määttänen et al., 2010; Figure 1).

Newly synthesized proteins in the ER are subjected to a rigorous quality control system and misfolded proteins are retrotransported back into the cytoplasm to be degraded by the ubiquitin-proteasome system. GRP78 associates with nascent chains immediately and for properly folded proteins, transiently upon synthesis. However, its association with misfolded or mutant proteins is prolonged (Sörgjerd et al., 2006). This prolonged association might be a signal for degradation of the bound protein (Petrova et al., 2008). The multistep process of ERAD, is initiated by GRP78 and other ER-resident chaperones that recognize the misfolded protein. Together, these chaperones facilitate deglycosylation and disassembling of misfolded proteins. The chaperones drive substrates to the translocon channel where they are pulled out of the membrane by a complex of proteins with ATPase activity. The emerging substrate is most likely ubiquitinated and addressed to the proteasome for degradation (review in Printsev et al., 2016; Figure 1). ERAD in combination with the ubiquitin-proteosome system (UPS) is thought to be the mechanism for quality control in long-lived cells such as neurons; hence, GRP78 is likely a critical component of the endogenous neuroprotective program.

Evidence from studies carried out in yeast indicates that when the ERAD system is saturated, macroautophagy removes both soluble and aggregated forms of unfolded proteins and dysfunctional organelles. Macroautophagy can be induced by various forms of cellular stress including nutrient or growth factor deprivation, hypoxia, reactive oxygen species, DNA damage, protein aggregates, damaged organelles, or intracellular pathogens (Klionsky et al., 2016). GRP78 plays a role in autophagic protein quality control, participating in the destruction of misfolded proteins in the cytosol. The autophagic process can be roughly divided into three steps: autophagosome formation, autophagosome-lysosome/late endosome fusion (autophagosome maturation), and degradation. The formation of autophagosomes necessitates the concerted and sequential action of autophagy related (ATG) proteins, originally identified in yeast (Itakura and Mizushima, 2010; Klionsky et al., 2016).

ATG proteins are regulated by conserved nutrient and energy-dependent signaling cascades that crucially involve the mammalian target of rapamycin (mTOR), a serine/threonine protein kinase belonging to the phosphatidylinositol kinase-related (PIKK) family, and AMP-activated protein kinase (AMPK). Starvation, amino acid deprivation, and growth factor withdrawal inhibit mTOR activity and lead to autophagy induction. AMPK is a major positive regulator of autophagy that is activated by low ATP availability (Kroemer et al., 2010). Both mTOR and AMPK control the cascade of events leading to the activation of the phosphatidylinositol 3-kinase class III (PI3KC3 also known as VPS34; Russell et al., 2013). PI3KC3, together with beclin 1, p150, and ATG14L, translocates to the initiation site of autophagosome formation (Matsunaga et al., 2010). At the ER, PI3KC3-mediated phosphatidylinositol 3-phosphate production (Axe et al., 2008; Hayashi-Nishino et al., 2009) fosters the formation of the phagophore. The phagophore sequesters cargo before closing in on itself to form the autophagosome. Phagophore expansion requires the conjugation of microtubule-associated protein 1A/1B-light chain 3 (LC3) to phosphatidylethanolamine, a process also called LC3 lipidation (Kabeya et al., 2000; Hamasaki et al., 2013). The LC3-positive autophagosome sequesters cytoplasmic material by binding to sequestosome 1 SQSTM1/p62. The autophagosome then fuses with an endosome or lysosome for cargo breakdown, and the degraded material is transported to the cytoplasm. SQSTM1/p62 binds LC3 and recruits proteins into autophagosomes for final degradation by lysosomal hydrolases.

GRP78 acts on the autophagic process at several points. Evidence for a role in the initiation and formation of the autophagosome is based on the finding that GRP78 overexpression increases autophagic signaling by stimulating AMPK (Cook and Clarke, 2012; Wen et al., 2012; Figure 1). In addition, GRP78 can interact to VPS34 and GRP78 overexpression activates the Class III PI3K-mediated autophagy pathway (Li et al., 2015). When GRP78 expression is inhibited, AMPK signaling activation does not occur (Cook and Clarke, 2012) and formation of autophagosomes is blocked (Li et al., 2009), although GRP78 deficiency does not prevent LC3 lipidation. GRP78 also acts at the final steps of macroautophagy since GRP78 binds to misfolded proteins and to SQSTM1/p62 in cells under stress. GRP78 binding induces a conformational change in SQSTM1/p62 that favors cargo delivery into the autophagosome for its subsequent degradation into amino acids (Jin et al., 2014; Kim et al., 2014; Abdel Malek et al., 2015; Cha-Molstad et al., 2015, 2016). Thus GRP78 acts as a chaperone for aggregation-prone misfolded proteins leading to their degradation by macroautophagy.

Macroautophagy is a pro-survival mechanism activated within the cell under stressful conditions. As it does in macroautophagy, GRP78 has a role in another cytoprotective process, the unfolded protein response (UPR) as well GRP78 (Paschen, 2004). The UPR is well-conserved from yeast to mammalian cells. Impaired processing and folding reactions that lead to an accumulation of misfolded proteins or potentially toxic aggregates, ATP depletion, and disturbances in calcium homeostasis, produce ER stress and UPR activation. To cope with ER stress, UPR activation coordinates the increase in ER-folding capacity through a broad transcriptional upregulation of ER folding, lipid biosynthesis, and ERAD machinery components with a decrease in folding load through selective mRNA degradation and translational repression (Gardner et al., 2013). GRP78 orchestrates the UPR by functionally regulating three ER transmembrane proteins that act as the main effectors: inositol-requiring enzyme 1 (IRE1), activating transcription factor 6 (ATF-6), and protein kinase R-like endoplasmic reticulum kinase (PERK; Schröder and Kaufman, 2005; Wang and Kaufman, 2016). GRP78 binds to IRE1, PERK, and ATF6 in unstressed cells and dissociates from these UPR sensors during acute ER stress (Bertolotti et al., 2000; Okamura et al., 2000; Shen et al., 2002; Figure 1).

IRE1 can also be directly activated by binding to unfolded proteins. Although ligand-induced oligomerization activates IRE1 (Shamu et al., 1994), GRP78 association stabilizes the inactive, monomeric form of IRE1 preventing its over response to low levels of ER stress (Korennykh et al., 2009; Pincus et al., 2010; Gardner and Walter, 2011). The RNase activity of IRE1 generates spliced mRNA encoding the X-box binding protein (XBP1), and XBP1 protein upregulates the expression of GRP78. ATF6 is cleaved by site 1 protease (S1P) and site 2 protease (S2P) to generate a p50-ATF6 fragment that has transcriptional activity. Upon cleavage, the p50-ATF6 fragment upregulates the expression of GRP78 through an ERSE in the promoter region of the *GRP78* gene as mentioned above. PERK has a kinase domain that phosphorylates the translation factor eIF2a, thereby suppressing most of the *de novo* protein synthesis during ER stress but stimulating the translation of certain mRNAs, including that encoding ATF4.

All of these processes are necessary to attenuate the accumulation of unfolded proteins during ER stress. IRE1 and ATF6 are especially critical in the prevention of ER stress-induced apoptosis via their upregulation of GRP78 expression (Gardner et al., 2013). Prolonged activation of IRE1 and CHOP can trigger apoptosis in cells under certain physiologic and pathophysiologic conditions (Szegezdi et al., 2006). In normal physiology, UPR-induced apoptosis may be a means to eliminate the few cells in an ER-stressed environment that remain uncorrected despite the actions of the UPR. Overexpression and antisense approaches in cell systems show that GRP78 can protect cells against cell death caused by disturbance of ER homeostasis (Morris et al., 1997; Yu et al., 1999; Jeon et al., 2016). Overexpression of GRP78 attenuates ER stress, both by enhancing protein folding and by helping to maintain IRE1, ATF6, and PERK in their inactive states (Bertolotti et al., 2000; Laybutt et al., 2007) and preventing *CHOP* induction to avoid apoptosis (Wang et al., 1996; Oyadomari and Mori, 2004).

#### GRP78 at the mitochondria and the mitochondria-associated ER membrane

GRP78 has also been observed in the mitochondria in association with co-chaperones known to be involved in calcium-mediated signaling between the ER and mitochondria that is important for bioenergetics and cell survival. ER stress and UPR signaling induce the overexpression of GRP78, which results in its mitochondrial localization. Sub-mitochondrial fractionation studies showed that GRP78 is mainly localized in the intermembrane space, inner membrane, and mitochondria matrix (Sun et al., 2006). GRP78 plays a direct role in controlling efflux of calcium ions from the ER by closing the Sec61 channel during protein translocation and in the absence of translocation (Hamman et al., 1998; Haigh and Johnson, 2002; Alder et al., 2005). In addition, upon calcium depletion from the ER via the inositol trisphosphate receptor IP_3_R, the calcium-sensitive co-chaperone sigma receptor 1 (Sig1R) dissociates from GRP78 and associates with IP_3_R, thereby protecting the otherwise unstable IP_3_R from ERAD and prolonging calcium signaling to the mitochondria (Hayashi and Su, 2007; Figure 1).

#### Secreted and cell-surface GRP78

Finally, GRP78 can be located at the plasma membrane where it is cytoprotective. In cultured cells, the ER stress agent, thapsigargin, actively promotes cell surface expression of GRP78, as the increase in cell surface GRP78 is several fold higher than the increase in intracellular GRP78 induced by thapsigargin (Zhang et al., 2010). Nonetheless, ER stress is not required for cell-surface localization of GRP78. Ectopic expression of GRP78 can induce its translocation in the absence of ER stress as indicated by the lack of CHOP induction. Moreover, deletion of the carboxyl-terminal ER-retention signal (KDEL) alters GRP78 relocation. This suggests that the KDEL retrieval system plays a significant role in regulating how much GRP78 leaves the ER.

Although GRP78 translocation have been studied mainly in cancer cell lines and have been found to be cell context-dependent (Tsai et al., 2015), there exist some common details for its mechanism of action. GRP78 can be translocated and anchored to the cell surface by binding to the ER-co-chaperone HTJ-1/MTJ-1 (Birukova et al., 2014; Figure 1). The translocation is promoted by accumulation of oxidized 1-palmitoyl-2-arachidonoyl-sn-glycero-3-phosphocholine (OxPAPC), a phospholipid that directly interacts with GRP78, induces membrane accumulation of the GRP78/HTJ-1 complex and its targeting to caveolin-enriched microdomains (Birukova et al., 2014). Once the complex is at the membrane, it activates Src/Fyn kinase leading to assembly of the PI3K complex and activation of mTOR and sphingosine-1-phosphate receptor 1. This in turn results in cortical actin cytoskeletal remodeling in endothelial cells. Thus, GRP78 regulates OxPAPC-mediated cytoskeletal remodeling.

In the plasma membrane, GRP78 functions as a signal-transducing receptor or co-receptor for soluble ligands such as α2-macroglobulin (α2-M; Misra et al., 2005b), tumor differentiation factor (Sokolowska et al., 2012), and vaspin (Nakatsuka et al., 2012). Other molecules that bind to GRP78 include glycosylphosphatidylinositol-anchored proteins, for example, T-cadherin (Philippova et al., 2008) and Cripto, the teratocarcinoma-derived growth factor (Shani et al., 2008), among others (Ni et al., 2011). In-depth details of activated downstream signaling due to these interactions have been extensively reviewed by Ni et al. (2011). The binding of GRP78 to most of these ligands activates the AKT/PI3K pro-survival pathway (Misra et al., 2004, 2006; Philippova et al., 2008; Figure 1). Soluble Cripto has also been shown to bind cell-surface GRP78/BiP initiating PI3K and MAPK signaling via Src activation (Gray and Vale, 2012) or binding directly to c-Src (Gu et al., 2015). Indeed, cell-surface GRP78 is also involved in cell-matrix adhesion by α1-integrin interaction and focal adhesion kinase (FAK) regulation. This interaction has been related to cell migration and invasion process, an effect partly mediated through its association with uPA–uPAR protease system (Li et al., 2013). The interaction with α1-integrin, considered also important for axonal regeneration, might be interesting to be further explored since GRP78 was found to promote neurite outgrowth *in vitro* (Satoh et al., 2000). Other interacting partners have been recently described that appear to be related to neurodegeneration which will be discussed below in other sections.

A recent study using a combination of biochemical, mutational, FACS, and single molecule super-resolution imaging approaches, reports that GRP78 mainly exists as a peripheral protein on plasma membrane via interaction with other cell surface proteins including glycosylphos-phatidylinositol-anchored proteins since it lacks a true transmembrane domain (Tsai et al., 2015). In addition, the authors discovered that cell-surface GRP78 expression requires its substrate binding activity but is independent of ATP binding.

Accordingly, GRP78 has also been observed as a secreted protein even in the human peripheral circulation (Delpino and Castelli, 2002). Secreted GRP78 can be found as well in the oviduct where apparently modulates sperm-zona pellucida binding (Marín-Briggiler et al., 2010). In a totally different context, the extracellular GRP78 has been proofed to have powerful immunomodulatory and anti-inflammatory properties by increasing IL-10 and reducing TNF-α (Corrigall et al., 2004; Panayi and Corrigall, 2014; Figure 1). This observation suggests that it would be relevant to determine such immunomodulatory property within the central nervous system.

#### Alternative variants of GRP78 in the cytoplasm

In addition to its localization in membrane-associated structures and organelles, GRP78 is observed in the cytoplasm. GRP78 can be relocated from the ER to the cytoplasm through several mechanisms: (i) via the ERAD pathway (Duriez et al., 2008), (ii) via a Bax/Bak-dependent change in membrane permeability produced during ER stress-induced apoptosis that allows luminal proteins to flow out (Wang et al., 2011), (iii) through *GRP78* alternative splicing of *GRP78* nuclear pre-RNA. The alternative processing results in retention of intron 1, which leads to an mRNA with an alternative translation initiation site and a premature stop codon that causes the loss of the ER signaling peptide in the encoded truncated isoform termed GRP78va (Ni et al., 2009; Figure 1).

## Lessons from cancer

Cancer cells are characterized by altered glucose metabolism, and the tumor microenvironment is marked by impaired blood flow and hypoxia, all of which can cause ER stress. GRP78 is involved in several aspects of cancer development including tumor survival and proliferation, chemoresistance, angiogenesis, and metastasis. Many tumor cells overexpress GRP78 on the outer plasma membrane. In addition, in different types of cancer, such as those of prostate, breast, and melanoma origins, abnormally high GRP78 expression is correlated with tumor resistance, greater risk for cancer recurrence, and an overall decrease in patient survival (reviewed in Pfaffenbach and Lee, 2011). Thus, GRP78 at the cell surface has been postulated to be a promising target for cancer therapeutics and a useful prognostic marker.

The utilization of knockdown and overexpression techniques and genetic mouse models has furthered our understanding of the role of GRP78 in cancer. In a transgene-induced endogenous mammary tumor model, GRP78 haploinsufficiency resulted in delayed tumor latency, decreased tumor proliferation, and increased apoptosis (Wang et al., 2010). Strikingly, in mice harboring bi-allelic conditional knockouts of both *GRP78* and *PTEN* in the prostate epithelium, prostate tumorigenesis was potently arrested, providing the first evidence that GRP78 is required for tumorigenesis driven by loss of *PTEN* and activation of the PI3K/AKT oncogenic pathway (Fu et al., 2008). Indeed, ligation of cell-surface GRP78 by antibody slowed growth rate and blocked PI3K/AKT signaling (Misra and Pizzo, 2010b).

Through formation of complexes with other proteins on the cell surface such as α2-M or Cripto, GRP78 is reported to mediate tumor cell signal transduction. Autoantibodies from serum of prostate cancer patients against a segment of GRP78 (Leu 98-Leu115) induces cell proliferation, suggesting that these antibodies serve as agonists of activated α2-M, which recognizes the same site of GRP78 (Gonzalez-Gronow et al., 2006). The interaction of α2-M with cell-surface GRP78 promotes cell proliferation by activating ERK1/2, p38 MAPK, and PI3K and enhances cell survival by inducing the AKT and NF-kB signaling cascades (Misra et al., 2004, 2006). In addition, in highly metastatic and invasive 1-LN prostate cancers, cell-surface GRP78 acts as a receptor for activated α2-M leading to activation of PAK-2, and together with LIMK and cofilin phosphorylation, increases motility enhancing metastasis (Misra et al., 2004, 2005a). Another pathway is triggered by binding to Cripto oncoprotein. The complex of Cripto and GRP78 enhances tumor growth via inhibition of TGF-β signaling. Furthermore, blockade of Cripto binding to cell-surface GRP78 by an antibody against the N-terminus of GRP78 inhibits oncogenic Cripto signaling and this involves the MAPK/PI3K and Smad2/3 pathways (Kelber et al., 2009). A commercial polyclonal antibody directed against the C-terminus of GRP78 was reported to induce apoptosis in melanoma cells (A375) and prostate cancer cells (1-LN, DU145) but not in the PC-3 prostate cancer cell line. GRP78 expression was undetectable on the surface of the PC-3 cells but was present on the other cell types (Misra et al., 2009). The proposed mechanism is that this antibody leads to suppression of Ras/MAPK and PI3K/AKT signaling (Misra et al., 2009; Misra and Pizzo, 2010a,b).

A different pathway has also been revealed recently. Katherine L. Cook and collaborators showed that GRP78 specifically inhibits *de novo* fatty acid synthesis in breast cancer cells and reduces mitochondrial β-oxidation through inhibition of mitochondrial carnitine palmitoyltransferase 1a (CPT1a), which catalyses the primary regulated step in overall mitochondrial fatty acid oxidation (Cook et al., 2016).

It has been suggested that GRP78 acts in concert to coordinate tumor cell growth to accommodate cancer cells to nutritional changes through facilitation of macroautophagy (Li et al., 2015). In agreement, one study showed that functional blockade of the proteasome induces GRP78, promoting autophagosome formation and enhancing myeloma survival (Abdel Malek et al., 2015). In tumor cells this activation can lead to autophagic degradation of IκB kinase, which caused inactivation of NF-κB pathway, an important mediator of apoptotic signaling.

The alternative cytosolic form, GRP78va is also important in tumorigenesis. This isoform is overexpressed in leukemic cells and leukemia patient samples. In the cytosol, GRP78va may associate with P58(IPK), which acts as inhibitor of PERK during UPR, antagonizing it and increasing cell survival under ER stress (Rutkowski et al., 2007). This study suggested that GRP78va has the potential to influence survival of cancer cells in adaptation to ER stress through modulating UPR signaling.

In summary, tumor cells use GRP78 to orchestrate the stimulation of processes such as macroautophagy, to combat the presence of reactive oxygen species (ROS), and to activate pro-survival signaling pathways.

## GRP78 in neurodegenerative processes

Age-related neurodegenerative diseases are commonly associated with the accumulation of misfolded and aggregated proteins and the presence of oxidative stress, calcium dysregulation, and mitochondrial dysfunction, particularly at the mitochondria-associated ER membrane (MAM). Neurodegenerative disorders, such as Alzheimer's disease (AD), Parkinson's disease (PD), amyotrophic lateral sclerosis (ALS), and prion-related diseases, have different clinical manifestations, but all present common events that also occur in neurodegenerative processes triggered by brain ischaemia or trauma. Aging, which is a risk factor for most neurodegenerative diseases, is accompanied by decreases in activity of several endogenous neuroprotective mechanisms that certainly may contribute to their etiopathogenesis.

### GRP78 in Alzheimer's disease

AD is a neurodegenerative disease characterized by cognitive alterations and memory loss. Early-onset cases of autosomal-dominant familial AD (FAD) are often caused by mutations in the genes encoding amyloid beta precursor protein (*APP*) or presenilin proteins (*PS1, PS2*). Aspartyl proteases PS1 and PS2 are components of the γ-secretase complex that, together with β-secretase, process APP to produce amyloid-β peptides (Aβ) of 40 and 42 amino acids (Aβ_40_, Aβ_42_). Hallmark lesions in AD are amyloid plaques and neurofibrillary tangles, both arising from protein misfolding. In plaques there is an abnormal increase in the Aβ_42_:Aβ_40_ ratio, whereas neurofibrillary tangles are composed of the aberrantly phosphorylated tau protein (Mattson, 1994).

The bulk of immature APP associates with GRP78 in the ER. GRP78 facilitates correct folding of APP and modulates intracellular APP maturation and processing (Yang et al., 1998; Kudo et al., 2006). Under ER stress, overexpression of GRP78 retains APP in the early secretory compartments resulting in a reduction of Aβ generation because β/γ-secretase activity itself is thought to be located in late secretory compartments, such as the Golgi apparatus and endo-lysosomal system (Kudo et al., 2006). In other way, GRP78 is a key player in APP processing also through ERAD. Some authors have found that another ER-resident protein dnj-27 (the ortholog of mammalian ERdj5), which works as an enhancer of ERAD together with GRP78 and EDEM, protects against the aggregation of both Aβ and α-synuclein (α-syn), involved in PD pathogenesis, in *C. elegans* (Muñoz-Lobato et al., 2014).

GRP78 levels are two-fold higher in AD temporal cortex and hippocampus compared to non-demented control cases as shown by immunohistochemistry. This increase was found in neurons in AD brains that were still healthy and that do not co-localize with neurofibrillary tangles indicating that GRP78 overexpression may slow down neurodegeneration (Hoozemans et al., 2005). Intriguingly, in the triple transgenic mice bearing FAD-linked mutations in APP and presenilins (3xTg-AD), which serve as an AD model, GRP78 levels are increased only by 1.5–2-fold in 2 month-old 3xTg-AD mice compared to controls, and this increase is associated with the presence of accumulated toxic Aβ peptide (Soejima et al., 2013). It is remarkable that this level of overexpression of GRP78, reported *in vivo* in this animal model and similar to those observed in post-mortem human AD tissue, is minor compared to the levels induced by ER stress (e.g., by using tunicamycin) in a wild-type animal, which can be more than 3-fold in several tissue types (Li et al., 2012; Galán et al., 2014). This observation suggests that the degree of GRP78 level increased in AD models and human AD neurons might be insufficient to cope with sustained ER stress. This observation is supported by age-related difficulties for GRP78 increase after ER stress as described further down in the aging section. In addition, it would be interesting to know where GRP78 is located within the neurons in AD tissues, as its functions depend on localization as discussed above. Importantly, the extracellular chaperone α_2_-M, a ligand of GRP78 at the plasma membrane, is co-localized with plaques in AD (Yerbury and Wilson, 2010), and it has been shown both to protect cells from Aβ toxicity and to favor Aβ removal from the brain (reviewed in Yerbury and Wilson, 2010). It is likely that some of these beneficial effects occur through the intervention of GRP78, although this has not been demonstrated yet.

Tau hyperphosphorylation is another pathological hallmark in AD brain and other Tauopathies. In a recent study, it was found that overexpression of GRP78 induced tau hyperphosphorylation via activating glycogen synthase kinase-3β (GSK-3β), an important tau kinase in AD brain, and increased the association with tau and GSK-3β. This was concurrent with SIL1 down regulated expression (Liu et al., 2016). However, when the authors forced the expression of both proteins prevented ER stress-induced tau hyperphosphorylation and GSK-3β activation suggesting the importance of ATP binding activity for beneficial effects promoted by GRP78.

In addition to APP processing, other abnormalities have been found associated to AD pathology where GRP78 can have also an opportunity for neuroprotection. Calcium level is dysregulated in AD brains, although its role in pathology is not well-understood. Calcium signaling may act even upstream of APP processing, as elevations in Ca^2+^ can increase production of oligomeric Aβ peptides (Itkin et al., 2011). Indeed, stabilizing ER calcium with dantrolene, a ryanodine receptor antagonist, restores normal synaptic function and plasticity and reduces amyloid load in the brains of 3xTg AD mice and knock-in FAD mice (reviewed in Frazier et al., 2017). A recent review by Area-Gomez (Area-gomez and Schon, 2016) proposed that the pathogenesis of AD might be mediated by increased ER-mitochondrial communication, which may cause aberrant increases in calcium trafficking between the two organelles, unusual phospholipid profiles, perturbed cholesterol homeostasis, changes in mitochondrial function and morphology, and an increased Aβ_42_:Aβ_40_ ratio. In particular, the authors argue that the altered ER membrane topology at the MAM in AD could explain the shift in the location of the γ-secretase cleavage toward Aβ_42_. In this regard, GRP78 localized at the MAM might have an important role in neuroprotection as a calcium binding protein.

One mechanism through which Aβ peptides cause cytotoxicity is by production of ROS via facile copper-redox cycling (Barnham et al., 2004), which can, in turn, result in oxidative damage to neuronal proteins and lipids (Mark et al., 1997). Imbalances in ROS production and detoxification are strongly implicated in AD neurodegeneration, as reflected by cerebral elevations in oxidized lipids and proteins (Sayre et al., 1997; Greilberger et al., 2008). According to recent studies revealing important roles of GRP78 in regulation of lipid content and inhibition of lipotoxicity resulting from lipid peroxidation and ROS generation (Cook et al., 2016) it is possible that overexpression of GRP78 can have neuroprotective properties against ROS as well.

Finally, sporadic AD (SAD) comprises the vast majority of AD cases. Mutations in the gene encoding apolipoprotein E (ApoE), particularly the *ApoE*ε*4* allele, are the strongest genetic risk. ApoEε4 promotes transient membrane cholesterol loading, which increases Aβ_42_ secretion and its accumulation in plaques in patients with AD and in cognitively normal people (reviewed in Sato and Morishita, 2015). Cholesterol and phospholipids have been shown to modulate the activity of APP-related secretases (Di Paolo and Kim, 2011). ER function is also affected by lipid composition and lipid biosynthetic enzymes (Lagace and Ridgway, 2013). Exogenous expression of GRP78 by adenoviral administration reduces liver lipogenesis by inhibiting activation of the central lipogenic regulator, the sterol regulatory element-binding protein 1c, SREBP1-c (Kammoun et al., 2009). Further, supporting the hypothesis that GRP78 modulates lipid metabolism, GRP78 heterozygous mice are resistant to obesity when placed on a high fat diet (Ye et al., 2010). Overexpression of GRP78 reduces the expression of lipogenic genes and plasma triglycerides and rescues the levels of the ER-processed ABCG5-G8 heterodimer transporter of cholesterol in the liver of obese mice lacking the receptor of leptin (db/db mice; Wang Y. et al., 2015). The mechanisms by which GRP78 functions in lipid and cholesterol management are far from clear, particularly in the brain, in light of these results in other tissues, it would be very interesting to further investigate its involvement in the lipid-related pathophysiology of neurodegenerative diseases such as AD.

### GRP78 in Parkinson's disease

Parkinson's disease is an idiopathic movement disorder characterized by the loss of dopaminergic neurons in the substantia nigra pars compacta (SNc) and the presence of Lewy bodies. Lewy bodies are distinct protein inclusions composed of aggregated α-syn. Studies on post-mortem brain samples have revealed immunoreactivity for UPR activation markers (Bellucci et al., 2011). Indeed, α-syn induces ER-stress and activates the UPR pathway in dopaminergic neurons in the SNc (Gorbatyuk et al., 2012).

In cell and animal models of α-syn accumulation, there is evidence that GRP78 forms a complex with α-syn (Bellucci et al., 2011; Colla et al., 2012; Gorbatyuk et al., 2012). Interestingly, both the level and localization of GRP78 are altered in different models of PD. For instance, in a rabbit model of PD, it has been demonstrated that GRP78 translocates from the ER to the nucleus and cytosol in response to treatment with MPP+, which causes a marked reduction in Tyrosine Hydroxylase-positive cells in the SNc (Ghribi et al., 2003). In cultured neurons, extracellular α-syn binds to GRP78 located at the cell surface, triggering a signaling cascade leading to cofilin 1 inactivation and stabilization of microfilaments, thus affecting morphology and dynamics of actin cytoskeleton. Inactivation of cofilin 1 and stabilization of actin cytoskeleton also occurs in fibroblasts derived from PD patients, suggesting that extracellular GRP78 might be the responsible. Dysregulation of actin turnover has been shown to lead to deficits in synaptic function that normally precede neurodegeneration in PD models. In addition, the interaction with extracellular α-syn renders GRP78 sequestered and clustered at the cell surface, which impedes its proper recycling toward the ER and results in a virtual depletion from the ER. Accordingly, overexpression of GRP78 was found to be neuroprotective, through a mechanism that involves decreases in the levels of UPR target genes, preventing the loss of dopaminergic neurons and dopamine in the SNc (Ni et al., 2011).

### GRP78 in amyotrophic lateral sclerosis

ALS is a progressive neurodegenerative disease, involving the selective degeneration of motoneurons in the spinal cord, most of the brainstem, and the cerebral cortex. Many different mutations are associated with familial ALS, but all lead to protein misfolding and aggregation. These mutations are in genes encoding superoxide dismutase 1 (SOD1), TAR DNA-binding protein 43-KDa, FUS, and other proteins. SOD1 aggregates have been observed in patients with sporadic ALS (Ezzi et al., 2007; Chattopadhyay et al., 2008; Bosco et al., 2010). Mutant SOD1 aggregates, but not wild-type SOD1, forms high molecular weight species that interact with GRP78 as observed in microsomal fractions of spinal cords derived from mouse models of ALS (Kikuchi et al., 2006).

Saxena's group investigated the pattern of expression of the ER folding network in vulnerable and resistant motoneurons and found that the ER folding network has a relevant role in ALS (Maharjan and Saxena, 2016). Remarkably, the knock-in mice that express mutant GRP78 lacking the KDEL sequence have age-related motor problems concomitant with loss of selective vulnerable motoneurons and aggregation of wild-type SOD1 reminiscent of ALS symptoms (Bosco et al., 2010; Jin et al., 2014). Several co-chaperones of GRP78, such as SIL1 and Sig1R, are important in ALS. SIL1 is mostly expressed in resistant motoneurons, suggesting it is involved in neuroprotection. Accordingly, SIL1 deficiency enhances ALS pathology, whereas SIL1 overexpression affords significant neuroprotection related to improved ER proteostasis and reduced SOD1 aggregation (reviewed in Rozas et al., 2017). Chronic treatment with PRE084, an agonist of Sig1R lead to increase neuroprotection of motoneurons in a mouse model of ALS (Mancuso et al., 2012). For all these reasons, it is possible that overexpression of GRP78 would mediate neuroprotection in ALS patients.

### GRP78 in prion-related diseases

Human prion diseases are rare, rapidly progressive, invariably lethal neurodegenerative diseases, symptomatically characterized by severe memory impairment and a general decline in cognitive functions, which may include motor, linguistic, executive, and social skills (Wadsworth et al., 2003). Most often, human prion diseases have a sporadic etiology [e.g., sporadic Creutzfeldt-Jakob disease (sCJD)], but hereditary (e.g., fatal familial insomnia and Gerstmann-Sträussler-Scheinker syndrome), and infectiously acquired [e.g., iatrogenic CJD, kuru, and variant CJD (vCJD)] forms of the disease also exist. Prion diseases have also extensively been described in animals; these include bovine spongiform encephalopathy (BSE) in cattle and scrapie in sheep.

At the neuropathological level, human prion diseases are characterized by the accumulation of pathological prion protein (PrP^Sc^), neuronal loss, astrogliosis, and spongiosis. During human prion disease progression, normal prion protein (PrP^C^) is converted into insoluble, β-sheet rich PrP^Sc^ aggregates. Once formed this pathological PrP^Sc^ conformer ensures conversion of native PrP^C^ into PrP^Sc^ and propagation of pathology to neighboring cells (reviewed in Wadsworth et al., 2003). One study reported increased expression of GRP78 and several other ER chaperones in post-mortem brain samples of sCJD and vCJD patients, although signal was not compared to controls (Hetz et al., 2003). In brain tissue samples from animals naturally infected with BSE, GRP78 is upregulated only by up to 2.3-fold (Tang et al., 2010). Increases in UPR markers such as GRP78 are thought to be an attempt of the neurons to cope with ER stress and are essentially markers of neuroprotective processes as mentioned. In a recent study, Jin and collaborators showed that GRP78 interacts transiently with PrP^c^ in the ER, in agreement with its involvement in the folding of nascent PrP^c^ polypeptides (Jin et al., 2000). GRP78 might remain associated for an extended period of time with some isoforms of mutant PrP causing its subsequent retrotranslocation for proteasomal degradation and so preventing the formation of homo-aggregates (Jin et al., 2000). It will be interesting to determine whether boosting GRP78 expression further will lead to neuroprotection as was demonstrated for another chaperone GRP58 (Hetz et al., 2005).

### GRP78 in neurodegenerative processes after ischemia or trauma to the nervous system

Neurodegeneration is a secondary event after traumatic brain injury and ischaemia. Ischemic preconditioning (IPC) is a sublethal ischemic episode that engages endogenous cytoprotective mechanisms to protect cells from subsequent severe ischemia (Zhang et al., 2015). As suggested by researchers in the field, uncovering the mechanisms of brain ischemic preconditioning might lead to the development of effective treatments for ischemic cerebrovascular disease that could be exploited therapeutically. Several studies have observed that IPC leads to upregulation of GRP78, which activates autophagy. Accordingly, specific suppression of GRP78 with pharmacological and genetic approaches inhibits autophagic activation and abolishes ischemic tolerance (reviewed in Zhang et al., 2015).

Overexpression of GRP78 is important for protection of astrocytes after ischemic injury as it reduces the flux of Ca^2+^ from the ER to the mitochondria, increases Ca^2+^ uptake capacity in isolated mitochondria, reduces free radical production, and preserves respiratory activity and mitochondrial membrane potential after stress (Ouyang et al., 2011).

After trauma, it has been demonstrated that GRP78 plays a relevant role. After abrupt proximal axotomy or avulsion of the nerve root, a retrograde neurodegenerative process occurs in spinal motoneurons. In contrast to root avulsion, after distal axotomy, motoneurons can engage signaling pathways that allow them to survive and regenerate. In these conditions, GRP78 is downregulated during neurodegenerative processes but overexpressed in the regenerative condition (Penas et al., 2009, 2011a). Indeed, forced expression of GRP78 or pharmacological activation of its co-chaperone Sig-R1 in a root avulsion model leads to neuroprotection (Guzmán-Lenis et al., 2009; Penas et al., 2011a,b). These observations suggested that GRP78 plays a relevant role activating endogenous neuroprotection and that its effects can be mimicked to exert neuroprotection in different conditions.

### GRP78 during aging

A commonality in neurodegenerative diseases is that the UPR is not correctly activated. In *ex vivo* human diseased brain tissue and *in vivo* models, there is significant depletion of ER molecular chaperones involved in the UPR despite ER stress (Lee et al., 2010; Gorbatyuk et al., 2012; Drake, 2015). Although, the mechanisms that underlie UPR dysfunction are unclear, aging might be a determinant factor. It has been reported that during aging, the quality control mechanism becomes inefficient since ER chaperones are less responsive to ER stress, as evidenced by decreased levels and activities of ER chaperones in aged tissue (Nuss et al., 2008). This defect has been attributed to increased oxidation of several key ER chaperones (Rabek et al., 2003), which would agree with the mitochondrial free radical theory of aging (Cadenas and Davies, 2000).

In particular, a reduction in GRP78 levels has been observed during aging and throughout progression of degenerative disorders (Paz Gavilán et al., 2006). Old mice (20–24 months old) have 20% less GRP78 ATPase activity than young mice (3–5 months old), which is consistent with a 2-fold higher level of GRP78 carbonylation in old mice. Such findings support the hypothesis that loss of ER or other cellular functions, often seen in age-related diseases, is caused by the life-long accumulation of oxidative damage to key proteins (Nuss et al., 2008; Salganik et al., 2015). Another study reported that there was about 73% less *GRP78* mRNA in old (900 days old) compared to young (21 days old) rats, suggesting that loss of GRP78 activity and the associated physiological declines occur at both the protein and transcript levels (Erickson et al., 2006). This suggests that the loss of GRP78 function could be a predisposing factor for neurodegenerative disorders associated with age (Brown and Naidoo, 2012).

A decrease in macroautophagy with age has also been reported in a variety of systems (Martinez-Lopez et al., 2015). The exact mechanisms by which protein aggregation contributes to neuronal degeneration remain to be fully elucidated; however, accumulating evidence suggests that defects in autophagy-related pathways contribute substantially to premature aging (Rajawat et al., 2009) and neurodegeneration (Ravikumar et al., 2004). Indeed, landmark studies have demonstrated that enhancing autophagy confers a protective effect in AD, PD, and Huntington's disease (reviewed in Ntsapi and Loos, 2016), whereas genetic suppression of basal autophagy causes neurodegeneration (Hara et al., 2006; Komatsu et al., 2006).

Successful and precise targeting of the autophagy process in the clinical setting has thus far not been accomplished, but it would be very interesting to know whether restoring GRP78 levels after ER stress in an aged-brain improve autophagy efficiency, reduces the extent of mitochondria dysregulation and protein aggregation.

## Concluding remarks

GRP78 or BiP is a very important protein. It has a relevant role to promote survival in tumor cells by activating potent endogenous cytoprotective mechanisms. Regarding these lessons, it is possible that engaging the same mechanisms in the nervous system this would be capable to cope with multiple stressful situations in the course of a disease. Multifunctional GRP78 can elicit neuroprotection by attenuating ER stress, managing misfolded proteins to avoid its accumulation, inducing macroautophagy, buffering calcium unbalance, facilitating mitochondria-ER crosstalk and activating pro-survival signaling pathways. Thus, GRP78 is an excellent target to take into consideration for neuroprotective therapeutical strategies targeting specifically neurons to avoid any putative undesirable side effect although GRP78 itself is not proto-oncogenic.

## Author contributions

CC has performed all the tasks necessary to carry out this review.

### Conflict of interest statement

The author declares that the research was conducted in the absence of any commercial or financial relationships that could be construed as a potential conflict of interest.

## Abbreviations

GRP78: 78-kDa glucose-regulated protein
UPR: unfolded protein response
ER: endoplasmic reticulum
PI3K: phosphatidylinositol 3-kinase
Hsp70: heat shock protein-70
ERSE: ER stress response element
CREB: cAMP responsive element CRE-like
IRES: internal ribosome entry sequence
NSAP1: NS1-associated protein
miRNA: microRNA
ERdj: ER resident J-domain co-chaperones
ERAD: endoplasmic-reticulum-associated protein degradation
AMPK: AMP-activated protein kinase
IRE1: inositol-requiring enzyme 1
ATF: activating transcription factor
PERK: protein kinase R-like endoplasmic reticulum kinase
XBP1: X-box binding protein
Sig1R: sigma receptor 1
IP_3_R: inositol trisphosphate receptor
KDEL: carboxyl-terminal ER-retention signal
OxPAPC: oxidized 1-palmitoyl-2-arachidonoyl-sn-glycero-3-phosphocholine
α2-M: α2-macroglobulin
CPT1a: carnitine palmitoyltransferase 1a
ROS: reactive oxygen species
ApoE: apolipoprotein E
SREBP1-c: sterol regulatory element-binding protein 1c
MAM: mitochondria-associated ER membrane
SNc: substantia nigra pars compacta
SOD1: superoxide dismutase 1
AD: Alzheimer's disease
sCJD: sporadic Creutzfeldt-Jakob disease
vCJD: variant CJD
BSE: bovine spongiform encephalopathy
PD: Parkinson's disease
PrP^Sc^: pathological prion protein
IPC: ischemic preconditioning
PrP^C^: prion protein
ALS: amyotrophic lateral sclerosis
FAD: autosomal-dominant familial AD
PS: presenilin
APP: amyloid beta precursor protein
Aβ: amyloid-β peptides
α-syn: α-synuclein.
